# Long-term immune responses in patients with confirmed novel coronavirus disease-2019: a 9-month prospective cohort study in Shanghai, China

**DOI:** 10.1186/s12879-022-07173-0

**Published:** 2022-03-10

**Authors:** Xiaohuan Gong, Peng Cui, Huanyu Wu, Hao Pan, Zheng Teng, Fang Yuan, Shenghua Mao, Dechuan Kong, Ruobing Han, Xue Zhao, Yaxu Zheng, Wenjia Xiao, Yiyi Zhu, Qiwen Fang, Sheng Lin, Bihong Jin, Ruilin Chu, Chenyan Jiang, Xiao Yu, Qi Qiu, Yihan Lu, Weibing Wang, Chen Fu, Xiaodong Sun

**Affiliations:** 1 Department of Epidemiology, School of Public Health, Fudan University, Shanghai, China; 2 Department of Infectious Disease Control and Prevention, Shanghai Municipal Center for Disease Control and Prevention, Shanghai, China; 3 Department of Microbiology, Shanghai Municipal Center for Disease Control and Prevention, Shanghai, China; 4 Shanghai Municipal Center for Disease Control and Prevention, Shanghai, China; 5 Ministry of Education Key Laboratory of Public Health Safety, Fudan University, Shanghai, China

**Keywords:** Covid-19, SARS-CoV-2, Cohort study, Antibodies, Immunity

## Abstract

**Background:**

The duration of antibodies against SARS-CoV-2 in Covid-19 patients remains uncertain. Longitudinal serological studies are needed to prevent disease and transmission of the virus.

**Methods:**

In 2020, 414 blood samples were tested, obtained from 157 confirmed Covid-19 patients, in a prospective cohort study in Shanghai.

**Results:**

The seropositive rate of IgM peaked at 40.5% (17/42) within 1 month after illness onset and then declined. The seropositive rate of IgG was 90.6% (58/64) after 2 months, remained above 85% from 2 to 9 months and was 90.9% (40/44) after 9 months. Generalized estimating equations models suggested that IgM (P < 0.001) but not IgG significantly decreased over time. Age ≥ 40 years (adjusted odds ratio [aOR] 4.531; 95% confidence interval [CI] 1.879–10.932), and cigarette smoking (aOR 0.344; 95% CI 0.124–0.951) were associated with IgG, and age ≥ 40 years (aOR 2.820; 95% CI 1.579–5.036) was associated with IgM. After seroconversion, over 90% and 75.1% of subjects were estimated to remain IgG-positive 220 and 254 days, respectively. Of 1420 self-reported symptoms questionnaires, only 5% reported symptoms 9 months after onset.

**Conclusions:**

In patients with a history of natural infection, anti-SARS-CoV-2 IgG is long-lived, being present for at least 9 months after illness onset. The long duration of natural immunity can mitigate and eliminate Covid-19 and the ongoing pandemic.

## Background

Coronavirus disease 2019 (Covid-19), caused by infection with severe acute respiratory syndrome coronavirus 2 (SARS-CoV-2), emerged in December 2019 and has since progressed rapidly worldwide [1, 2]. The World Health Organization (WHO) declared a Public Health Emergency of International Concern (PHEIC) in January 2020 and a pandemic in March 2020 [3, 4]. As of 9 January 2022, almost two years after the WHO declared the PHEIC, there were more than 304 million Covid-19 confirmed cases and over 5.4 million deaths worldwide [5]. Although Covid-19 has had an unprecedented impact on the world, understanding of this disease, especially of immunity after recovery, remains insufficient [6, 7].

Limited pre-existing immunity to SARS-CoV-2 is considered responsible for the explosive increase in the number of cases [6]. Vaccines developed against SARS-CoV-2 are expected to curb and control the Covid-19 pandemic [7, 8]. The duration of immune responses to SARS-CoV-2 infection is of crucial importance for both vaccination and treatment, and will affect public health and clinical strategies. Some findings raised concern that humoral immunity against SARS-CoV-2 may not be long lasting in persons with mild illness, who compose the majority of persons with Covid-19 [9, 10]. If initial infection reduces susceptibility to re-infection and if the duration of natural immunity is long, then infection would be delayed and suppressed [8]. Repeated cross-sectional sero-monitoring has found that antibody levels in the population last about 3 months [11], and a small-scale cohort study has found that seropositivity for IgG antiviral antibodies did not change significantly over 6 months, with 90% of subjects remaining positive [12]. Moreover, a large-scale study found that previous infection, resulting in antibodies to SARS-CoV-2, is associated with protection from reinfection for most people for at least 6 months [13].

Preliminary studies have yielded inconclusive results about the duration of antiviral antibodies and the ability of these antibodies to protect against SARS-Cov-2 [14], with the longest follow-up duration after Covid-19 onset being 6 months. The presence of antiviral antibodies can both prevent disease and onward transmission of the virus. However, the duration of antiviral antibodies in Covid-19 patients remains unclear, indicating the need for longitudinal serological studies. The present cohort study therefore assessed temporal changes in immunoglobulin G (IgG) and IgM antibodies against SARS-CoV-2 and antiviral immune responses for 9 months after illness onset in patients with confirmed Covid-19 in Shanghai, China, as well as identifying factors associated with these changes.

## Methods

### Design and participants

This prospective cohort study of patients in Shanghai discharged after having confirmed Covid-19 was started in March 2020. Patients were diagnosed, treated and discharged according to the Diagnosis and Treatment Guidelines for Novel Coronavirus Pneumonia issued by the National Health Commission of the People's Republic of China [15]. A suspected case was defined as any person with the clinical signs of Covid-19 and/or with an epidemiological history. Respiratory specimens (nasopharyngeal swab, pharyngeal swab, sputum) of suspected cases were collected and tested for SARS-CoV-2 by real-time reverse-transcriptase polymerase chain-reaction (rRT-PCR) assay. A confirmed case was any suspected case with respiratory samples testing rRT-PCR-positive for SARS-CoV-2. All confirmed case with Covid-19 received hospitalization treatment. The discharge criteria included a normal body temperature for 3 consecutive days, significant improvements in respiratory symptoms and lung imaging; and respiratory samples negative for SARS-CoV-2 on two consecutive samplings at least 24 h apart.

This study included all patients in Shanghai confirmed as having Covid-19 on December 10. Patients were excluded if they: (1) died before discharge, (2) had not been discharged or were discharged within 7 days, (3) were unable to be contacted, (4) were lost to follow-up, or (5) refused participation or lived outside Shanghai.

### Procedures and setting

Patients were contacted by telephone within 7 days after hospitalization discharge by trained healthcare workers of the corresponding community health centers. Participants who agreed to participate were interviewed within 1 month and at 1, 2, 3, 6, and 9 months after onset of illness. Patients who agreed were also followed-up at 4–5 and at 7–8 months. Follow-up consisted of sample collection, as well as a face-to-face interview, including administration of a structured questionnaire, at the outpatient clinic of each community health center or hospital. When face-to-face interviews could not be performed, questionnaires were administered over the telephone.

### Sample collection and laboratory assays

Blood samples were collected in clinical laboratories of each outpatient clinic, stored at 4 °C and sent to Shanghai Municipal CDC within 3 days. All tests of blood samples were performed in the laboratory of Shanghai Municipal CDC. IgM and IgG antibodies against SARS-Cov-2 were assayed using colloidal gold immunoassays kits (Innovita Co., Ltd, China) [16–18], which have been approved by the China National Medical Products Administration (approval No.: 202003400177) and the European Union (Cert. No.: EU208518) as in-vitro diagnostic medical devices, according to the manufacturer’s instructions. Blood samples were added onto the test pad, which was incubated flat at room temperature (10–30 °C) for 25 min, with the appearance of an IgM or IgG line indicated a positive result for IgM or IgG, respectively, whereas a negative test produces only the control line. Negative and weakly positive results were re-tested using colloidal gold immunoassay kits from another manufacturer (Vazyme Co., Ltd, China), approved by the China National Medical Products Administration (approval No.: 202003400239). Blood samples were also subjected to flow cytometry analysis (Roche.) to measure counts of peripheral lymphocytes (T cells, CD4^+^ T cells, CD8^+^ T cells and B cells) with fluorochrome-conjugated antibodies (BD Biosciences).

### Outcomes and measurements

The primary outcomes were IgM and IgG antibodies against SARS-CoV-2 in blood samples, as determined by to Prevention and Control Guidelines for Novel Coronavirus Pneumonia [19] issued by the National Health Commission of the People’s Republic of China. The secondary outcomes were cell counts per ml in blood samples of T cells, CD4+ T cells, CD8+ T cells and B cells.

Subject characteristics and factors were obtained from epidemiological investigations and patients’ medical records. Patients were divided into two age groups (0–39,40–89) or four age groups (0–24, 25–39, 40–59, 60–89), and two BMI groups, defined as underweight or normal (BMI ≤ 23.9 kg/m^2^), and overweight or obese (BMI ≥ 24.0 kg/m^2^) subjects. Potential factors incorporated into generalized estimating equations models included age group, gender, cigarette smoking (yes or no), alcohol drinking (yes or no), BMI group, abroad imported case (yes or no), involvement in a cluster (yes or no), duration from onset to admission (days), duration from admission to discharge (days), comorbidities (yes or no), clinical intensity (mild, normal, severe or critical), any symptoms (yes or no), fever (yes or no), pneumonia (yes or no), cough (yes or no), fatigue (yes or no), and first blood test (white blood cell count × 10^9^/L, leukomonocyte cell count × 10^9^/L). The clinical severity of a patient was divided into mild, normal, and severe according to clinical manifestations [15].

The acute phase was defined as the time between symptom onset and hospital discharge. Follow-up questionnaires included participants’ self-reported symptoms, whether newly occurring or persistent.

### Statistical analysis

Continuous variables were reported as medians and interquartile ranges (IQRs) and compared by the Kruskal–Wallis test. Categorical variables were reported as numbers and percentages and compared by the Cochran-Mantel–Haenszel test. Generalized estimating equations (GEE) models were employed to test the association between immune response and potential factors, considering the temporal impact of the repeated measurements, with or without a logic link according to the type of variables. Each serological result was regarded as interval-censored. Cumulative seroprevalence were estimated using the combined self-consistent EM algorithm and the iterative convex minorant algorithm (EMICM, Wellner and Zhan, 1997). Survival analysis outcomes, including seroconversion intervals and antibody duration, were compared using proportional hazards models and generalized log-rank test, specialized for interval censored data. All statistical analyses were performed using SAS version 9.4 (SAS Institute, Cary, NC), whereas figures were drawn using R version 4.0.4.

## Results

**Fig. 1:**
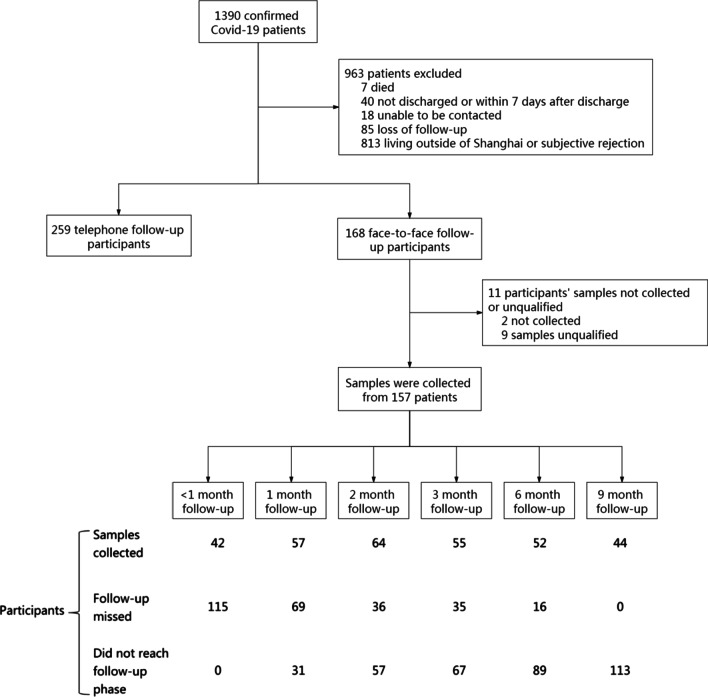
Flowchart of follow-up of patients with confirmed Covid-19 in this prospective cohort study

As of December 10, 2020, 1390 patients in Shanghai had been confirmed as having Covid-19. Of these, 963 patients were excluded, seven because they died before hospital discharge, 40 because they were not discharged or were within 7 days after discharge, 18 because they could not be contacted, 85 because they were lost to follow-up, and 813 because they refused to participate or lived outside Shanghai (Fig. 1). Of the remaining 427 discharged patients, 168 were followed-up face-to-face and 259 were followed-up by telephone. Blood samples were collected and assayed from 157 of the 168 patients followed-up face-to-face; these 157 patients were regarded as cohort patients in analysis. The rest 1233 patients were regarded as non-cohort patients.

### Characteristics of patients with confirmed Covid-19

The 157 cohort patients consisted of 91 (58.0%) males and 66 (42.0%) females, of median age 36.0 years. Eighty (52.0%) patients were underweight or normal (BMI ≤ 23.9 kg/m^2^), and 77 (48.0%) were overweight or obese (BMI ≥ 24.0 kg/m^2^). In addition, 65 (41.4%) were local residents, 61 (38.9%) were abroad imported cases and 108 (68.8%) had normal clinical intensity. The 1233 non-cohort patients included 762 (61.8%) males and 471 (38.2%) females, of median age 35.0 years. 419 (60.6%) were underweight or normal (BMI ≤ 23.9 kg/m^2^), and 272 (39.4%) were overweight or obese (BMI ≥ 24.0 kg/m^2^). In addition, 167 (13.5%) were local residents, 980 (79.5%) abroad imported cases and 67 (50%) had normal clinical intensity. Significant differences were observed between cohort and non-cohort patients in BMI group, residency, and clinical intensity (Appendix Table 4).

### Characteristics of participants in the cohort

The median duration from illness onset to final follow-up of the 157 patients in the study cohort was 122 [IQR 37, 272] days. During the follow-up period, the total seroconversion rates were 40.76% (64/157) for IgM and 88.54% (139/157) for IgG. The difference of seroconversion rates for IgM among 4 age groups was significant (*P* = 0.028), higher in 40–59 years age group (55.8%) and 60–79 years age group (42.9%). The difference of seroconversion rates for IgG among 4 age groups was significant (*P* = 0.020), higher in 40–59 years age group (97.7%) and 60–79 years age group (95.2%). The difference of pneumonia (*P* = 0.02) and fever (*P* = 0.001) among 4 age groups was significant, most fever (100.00%) and pneumonia (71.4%) in 60–79 years age group. The demographic, epidemiological and clinical characteristics of participants in the cohort are presented in Table 1.

**Table 1:** Demographic, epidemiological and clinical characteristics of participants in the study cohort

Characteristics	Cohort patients (n = 157)	IgM antibody^†^			IgG antibody^†^		
		IgM negative (n = 93)	IgM positive (n = 64)	*P* value	IgG negative (n = 18)	IgG positive (n = 139)	*P* value
Age (years)				0.028*			0.020*
0–24	29 (18.5)	23 (24.7)	6 (20.7)		7 (38.9)	22 (15.8)	
25–39	64 (40.8)	39 (41.9)	25 (39.1)		9 (50.0)	55 (39.6)	
40–59	43 (27.4)	19 (20.4)	24 (37.5)		1 (5.6)	42 (30.2)	
60–79	21 (13.4)	12 (12.9)	9 (14.1)		1 (5.6)	20 (14.4)	
Gender				0.975			0.428
Male	91 (58.0)	54 (58.1)	37 (57.8)		12 (66.7)	79 (56.8)	
Occupation				0.143			0.032*
Students/Children	30 (19.1)	23 (24.7)	7 (10.9)		7 (38.9)	23 (16.5)	
Retirees	28 (17.8)	16 (17.2)	12 (18.8)		1 (5.6)	27 (19.4)	
Civilian staff	53 (33.8)	31 (33.3)	22 (34.4)		8 (44.4)	45 (32.4)	
Other occupations	46 (29.3)	23 (24.7)	23 (35.9)		2 (11.1)	44 (31.7)	
Healthcare workers	3 (1.9)	2 (2.2)	1 (1.6)	0.792	0 (0.0)	3 (2.2)	0.530
Pregnant women	2 (3.0)	2 (5.1)	0 (0.0)		1 (16.7)	1 (1.7)	0.043*
BMI (kg/m^2^)				0.122			0.266
< 24.0	80 (52.0)	52 (57.1)	28 (44.4)		11 (64.7)	69 (50.4)	
≥ 24.0	74(48.1)	39 (42.9)	35 (55.6)		6 (35.3)	68 (49.6)	
Residence							0.045*
Within Shanghai	65 (41.4)	39 (41.9)	26 (40.6)	0.667	3 (16.7)	62 (44.6)	
Outside Shanghai	85 (54.1)	51 (54.8)	34 (53.1)		13 (72.2)	72 (51.8)	
Foreign born	7 (4.5)	3 (3.2)	4 (6.2)		2 (11.1)	5 (3.6)	
Cigarette smoking	18 (12.2)	11 (12.8)	7 (11.3)	0.784	5 (27.8)	13 (10.0)	0.031*
Alcohol drinking	45 (30.4)	25 (29.1)	20 (32.3)	0.678	4 (22.2)	41 (31.5)	0.422
Comorbidities							
Diabetes	7 (4.6)	6 (6.6)	1 (1.6)	0.149	0 (0.0)	7 (5.2)	0.340
High blood pressure	17 (13.5)	11 (15.1)	6 (11.3)	0.545	1 (7.7)	16 (14.16)	0.520
Heart disease	3 (2.0)	1 (1.1)	2 (3.2)	0.378	0 (0.0)	3 (2.2)	0.548
Abroad imported patient	61 (38.9)	37 (39.8)	24 (37.5)	0.774	10 (55.6)	51 (36.7)	0.124
Involved in a cluster	48 (30.6)	31 (33.3)	17 (26.6)	0.367	2 (11.1)	46 (33.1)	0.058
Clinical intensity							0.199
Mild	49 (31.2)	28 (30.1)	21 (32.8)	0.720	8 (44.4)	41 (29.5)	
Normal	108 (68.8)	65 (69.9)	43 (67.2)		10 (55.6)	98 (70.5)	
SARS-CoV-2 rPCR CT value in acute phase	29.0 [26.0, 33.6]	28.5 [21.5, 32.7]	31.2 [28.8, 34.3]	0.310	30.3 [29.0, 34.9]	27.2 [24.5, 31.8]	0.464
Duration from onset to admission, days	4.0 [2.0, 7.0]	4.0 [2.0, 7.0]	3.0 [1.5, 7.0]	0.731	2.0 [1.0, 4.0]	4.0 [2.0, 7.0]	0.058
Duration from admission to discharge, days	14.0 [10.0, 21.0]	14.0 [10.0, 21.0]	15.0 [10.0, 20.5]	0.750	13.0 [10.0, 23.0]	15.0 [10.0, 21.0]	0.816
Duration from onset to last follow-up, days	122 [37, 272]	104.0 [35.0, 263.0]	160.0 [23.0, 39.5]	0.207	66.5 [29.0, 176.0]	146.0 [43.0, 274.0]	0.047*
Duration from discharge to last follow-up, days	106 [13, 247]	81.0 [14.0, 237.0]	144.0 [12.5, 253.0]	0.376	36.5 [9.0, 162.0]	125.0 [14.0, 248.0]	0.047*
Symptoms during acute phase							
Any symptom^§^	120 (76.43)	70 (75.3)	50 (78.13)	0.680	11 (61.1)	109 (78.4)	0.105
Fever	107 (68.2)	63 (67.7)	44 (68.8)	0.894	8 (44.4)	99 (71.2)	0.022*
Highest body temperature	38.0 [37.7, 38.6]	38.2 [37.6, 38.8]	38.0 [37.7, 38.4]	0.289	38.2 [37.9, 38.8]	38.0 [37.6, 38.6]	0.512
Cough	51 (32.5)	34 (36.6)	17 (26.6)	0.190	6 (33.3)	45 (32.4)	0.935
Sputum production	14 (8.9)	10 (10.8)	4 (6.3)	0.332	1 (5.6)	13 (9.4)	0.596
Throat soreness	12 (7.6)	8 (8.6)	4 (6.3)	0.811	0 (0.0)	12 (8.6)	0.196
Fatigue	16 (10.2)	10 (10.8)	6 (9.4)	0.780	0 (0.0)	16 (11.5)	0.130
Pneumonia	69 (44.0)	38 (40.9)	31 ( 48.4)	0.349	7 (38.9)	62 (44.6)	0.647
First blood test							
White blood cell count (× 10^9^/L)	5.9 [4.7, 7.3]	5.9 [4.5, 7.3]	5.7 [4.8, 7.4]	0.728	7.2 [5.6, 8.0]	5.8 [4.5, 7.2]	0.024*
Leukomonocyte cell count (× 10^9^/L)	1.4 [1.0, 1.8]	1.5 [1.0, 2.2]	1.4 [1.1, 1.7]	0.059	1.7 [1.4, 2.6]	1.4 [1.0, 1.8]	0.038*
Leukomonocyte cell percentage (%)	26.1 [19.7, 32.4]	28.5 [20.0, 34.4]	23.7 [19.6, 30.0]	0.027*	27.9 [24.7, 33.4]	25.0 [19.7, 32.1]	0.436
Neutrophil count (× 10^9^/L)	4.6 [3.8, 6.4]	4.6 [3.9, 6.4]	5.0 [3.0, 29.0]	0.869	4.6 [4.6, 4.6]	4.6 [3.8, 6.4]	0.855
Neutrophil percentage (%)	62.5 [57.8, 71.2]	60.1 [55.2, 69.5]	65.7 [59.3, 71.9]	0.034*	60.7 [54.2, 66.6]	62.8 [57.8, 71.7]	0.327
C-reactive protein(mg/L)	8.7 [3.7, 15.1]	8.7 [3.0, 12.0]	8.8 [5.0, 17.6]	0.372	7.7 [5.0, 10.9]	8.7 [3.7, 15.6]	0.600

Continuous variables are reported as median [IQR], and categorical variables as number (percent)

*P < 0.05; **P < 0.01

^†^Ab positive defined as any follow-up sample testing positive after discharge. Ab negative defined as all follow-up samples testing negative after discharge

^§^Symptoms include fever, cough, sputum production, throat soreness, headache, dizziness, nasal congestion, runny nose, muscle soreness, joint pain, fatigue, shortness of breath, difficulty breathing, diarrhea, pneumonia, and conjunctivitis. Some symptoms are not shown due to small percentages

Median [IQR] age was significantly higher in patients positive than negative for antiviral IgM (40 [31.5, 55.0] years vs. 32.0 [25.0, 50.0] years, *P* = 0.011) Leukomonocyte cell percentage during the acute phase was significantly lower (*P* = 0.027), while neutrophil percentage was significantly higher (*P* = 0.034), in patients positive than negative for antiviral IgM.

Median age was also significantly higher in patients positive than negative for antiviral IgG (37.0 [29.0, 54.0] years vs. 25.0 [21.0, 32.0] years, *P* < 0.001). The percentage of cigarette smokers was significantly lower (10% vs. 27.8%, *P* = 0.031), and the percentage having fever during the acute phase significantly higher (71.2% vs. 44.4%, *P* = 0.022), among patients positive than negative for antiviral IgG. White blood cell (*P* = 0.024) and leukomonocyte cell (*P* = 0.038) counts during the acute phase were significantly lower in IgG-positive than in IgG-negative participants. Other characteristics, including gender, BMI, alcohol drinking, comorbidities, clinical intensity, and symptoms other than fever, did not differ significantly in patients positive and negative for antiviral antibody.

### Temporal changes in IgM and IgG antibody responses

**Fig. 2:**
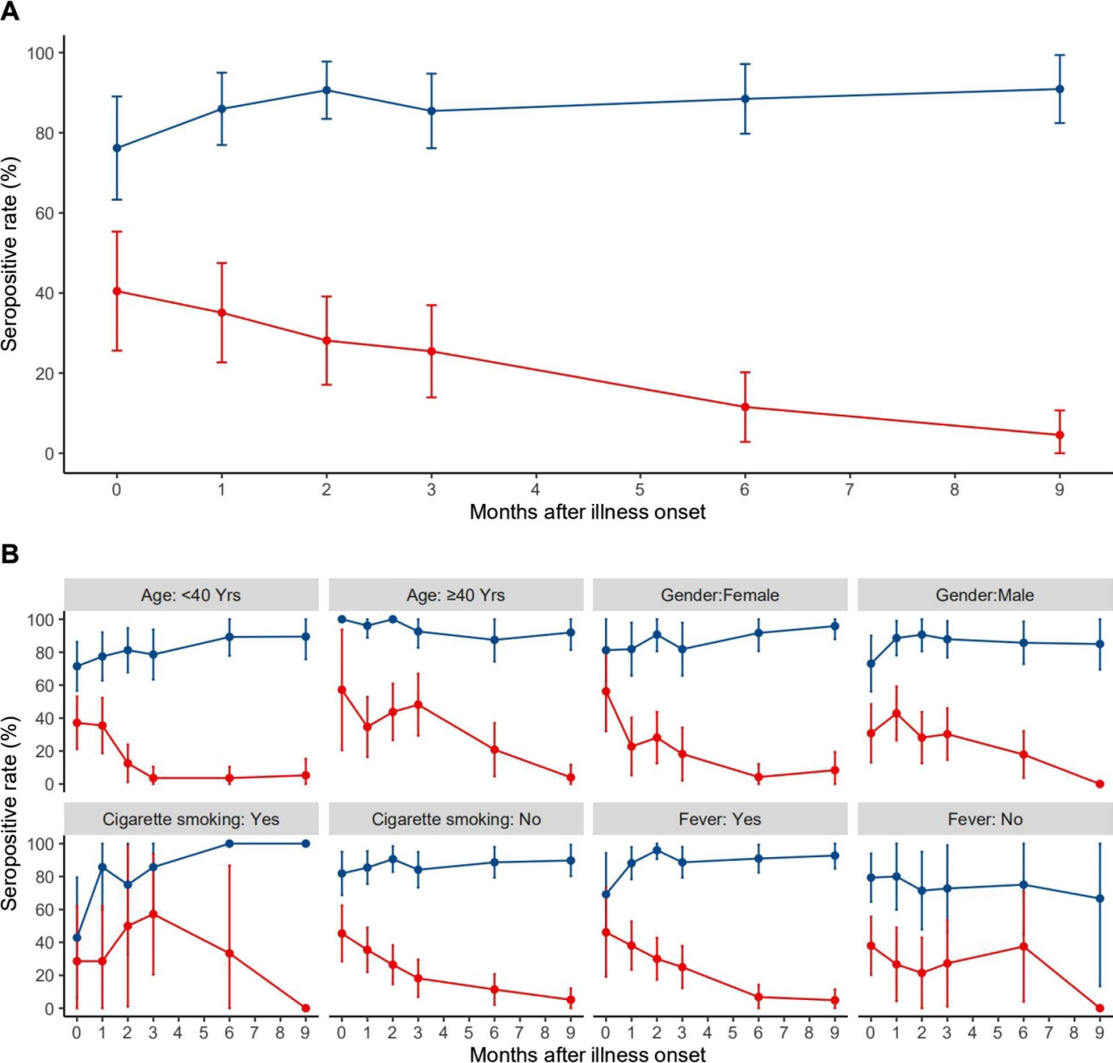
Temporal changes of seropositive rate of IgM and IgG antibodies against SARS-CoV-2 in the total cohort and in subgroups. (**A** Seropositive rate of IgM and IgG against SARS-CoV-2. **B** Seropositive rate of IgM and IgG against SARS-CoV-2 by factors. Blue line means IgG, and red line means IgM.)

From March to December 2020, 414 blood samples were obtained from the 157 patients in the study cohort and tested for antibodies against SARS-CoV-2. The seropositivity rate for antiviral IgM was maximal at 40.5% (17/42) within 1 month after illness onset, declining to 35.1% (20/57), 25.5% (14/55), 11.5% (6/52), and 4.55% (2/44) at 1, 3, 6 and 9 months, respectively (Fig. 2). The rate was higher at 7–8 months (23.1%, 3/13), perhaps due to the small number of samples assayed at that time points.

The seropositivity rate for antiviral IgG increased gradually during the first 2 months after illness onset, from 76.2% (32/42) within 1 month to 90.6% (58/64) after 2 months. This rate remained above 85% from 2 to 9 months, being 85.5% (47/55), 88.5% (46/52) and 90.9% (40/44) after 3, 6, and 9 months, respectively (Fig. 2). These rates were lower at 4–5 months (82.8%, 24/29) and 7–8 months (76.9%, 10/13), perhaps due to the relatively small number of samples assayed at those time points.

GEE model analyses with logit link of antiviral IgG seropositivity, which included two covariates (duration since disease onset and one potential factor), found that age ≥ 40 years (adjusted odds ratio [aOR] 4.531; 95% confidence interval [CI] 1.879–10.932, *P* = 0.001), cigarette smoking (aOR 0.344; 95% CI 0.124–0.951, *P* = 0.040), fever (aOR 2.479, 95% CI 1.078–5.698, *P* = 0.033), fatigue (aOR 7.508; 95% CI 1.671–33.734, *P* = 0.009), white blood cell count (aOR 0.812, 95% CI 0.686–0.963, *P* = 0.017), and leukomonocyte cell count (aOR 0.502, 95% CI 0.275–0.918, *P* = 0.025) were significantly associated with antiviral IgG. In contrast, age ≥ 40 years (aOR 5.165, 95% CI 1.469–18.151, *P* = 0.011) was the only factor associated with antiviral IgG on multivariate GEE analysis, including duration since onset and the above significant factors potential factors. GEE model analyses found that there was no tendency for IgG to decrease over time for up to 9 months after disease onset (Table 2).

**Table 2:** Association between IgG antibody and cohort patient characteristics in GEE model analyses

Factor	Model 1^†^		Model 2^#^	
	Adjusted OR (95%CI)	*P* value	Adjusted OR (95%CI)	*P* value
Age, 40-years vs. 0–39 years	4.531 (1.879–10.932)	0.001**	5.165 (1.469–18.151)	0.011*
Cigarette smoking	0.344 (0.124–0.951)	0.040*	0.366 (0.112–1.194)	0.096
Fever	2.479 (1.078–5.698)	0.033*	1.326 (0.458–3.842)	0.603
Fatigue	7.508 (1.671–33.734)	0.009**	5.585 (0.819–38.084)	0.079
First blood test				
White blood cell count (× 10^9^/L)	0.812 (0.686–0.963)	0.017*	0.85 (0.674–1.072)	0.169
Leukomonocyte cell count (× 10^9^/L)	0.502 (0.275–0.918)	0.025*	0.869 (0.41–1.843)	0.715

Generalized estimating equation (GEE) models with logit link functions for repeated measure were used. Only statistically significant results are shown

*P < 0.05; **P < 0.01

^†^Two dependent variables include duration from onset to follow-up (days) and one of the following factors, gender, age, cigarette smoking, alcohol drinking, BMI, abroad imported patient, involvement in a cluster, duration from onset to admission (days), duration from admission to discharge (days), comorbidities, clinical intensity, any symptom, fever, pneumonia, cough, fatigue, first blood test (white blood cell count (× 10^9^/L), leukomonocyte cell count (× 10^9^/L))

^#^Duration from onset to follow-up (days) and statistical significant factors in model 1 were included in model 2. Results of all included factors are shown in the table

GEE model analyses with logit link of antiviral IgM positivity including 2 covariates (duration since onset and one of potential factors), found that age ≥ 40 years (aOR 2.820, 95% CI 1.579–5.036, *P* < 0.001) was the only factor significantly associated with antiviral IgM. GEE model analyses found that IgM decreased significantly over time (*P* < 0.001).

### Seroconversion and duration of IgM and IgG antibodies

**Fig. 3:**
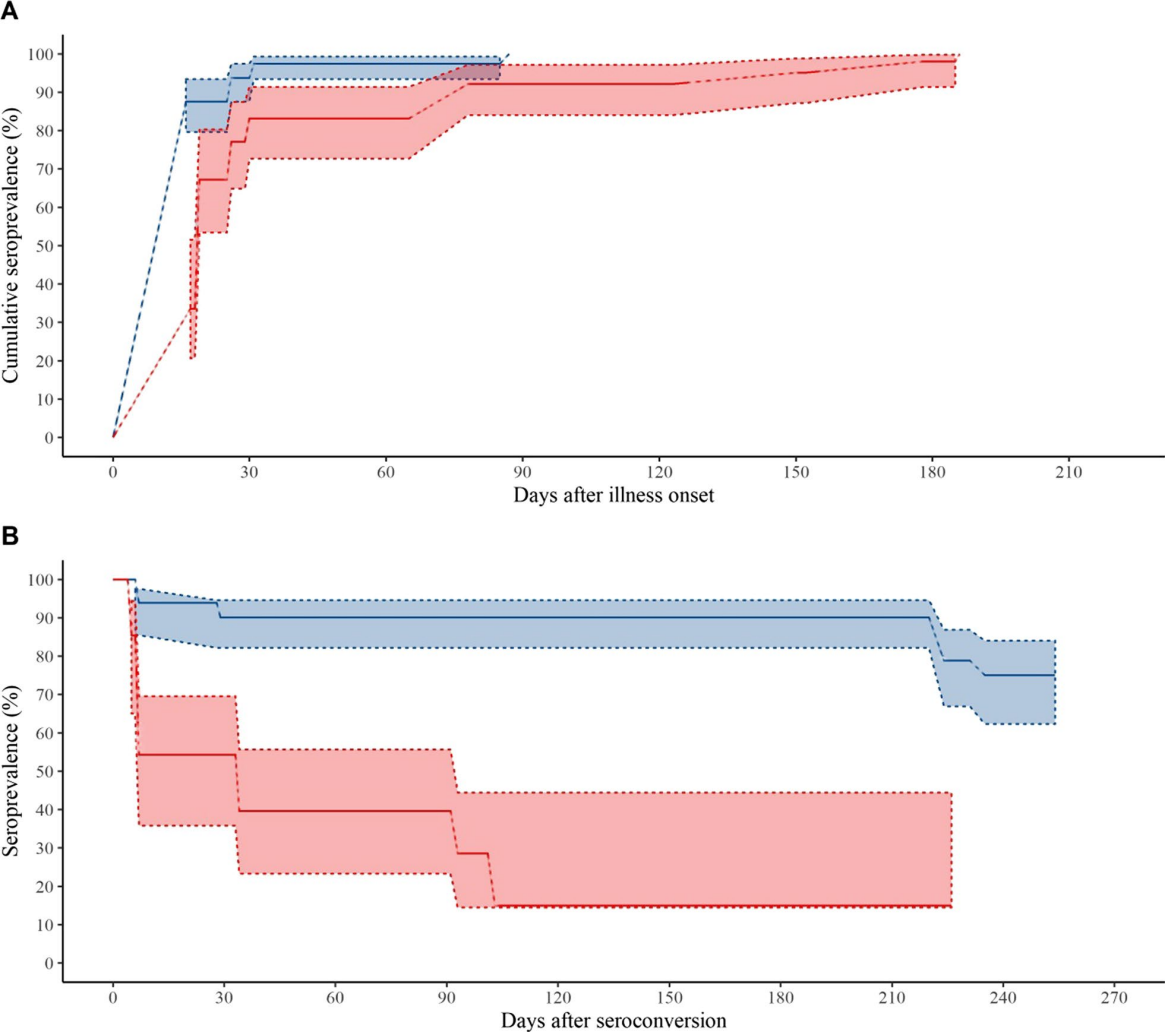
Seroconversion and duration of IgM and IgG antibodies against SARS-CoV-2. (**A** Seroconversion of IgM and IgG. **B** Duration of IgM and IgG. Blue line means IgG, and red line means IgM. The shaded areas represents the 95% confidence interval.)

The estimated cumulative seroprevalences of IgM and IgG are presented in Fig. 3A. The cumulative seroprevalences of IgM and IgG were 83.2% and 97.4%, respectively, 1 month after the onset of illness, showing that the cumulative seroprevalence of antiviral IgG was early and higher than the cumulative seroprevalence of antiviral IgM during the 1st month. Overall, the seroconversion of IgG was significantly more rapid than that of IgM (P < 0.001).

The estimated durations of antiviral IgM and IgG are presented in Fig. 3B. Among participants who developed antiviral IgG, 90.1% and 75.1% remained positive for these antibodies at 220 days (about 7 months) and 254 days (about 8 months), respectively. Among subjects who developed antiviral IgM, only 54.3%, 39.7%, and 14.9% remained positive at 33 days (about 1 month), 91 days (about 3 months), and 103 days (about 3.5 months), respectively. Overall, the duration of antiviral IgG was significantly longer than that of antiviral IgM (P < 0.001).

Univariate proportional hazards models found that abroad imported case (hazard ratio (HR) 6.331, 95% CI 1.56–26.63, *P* = 0.010) and fever (HR 0.450, 95% CI 0.204–0.993, *P* = 0.048) were significantly associated with IgG seroconversion time. Similarly, abroad imported case (HR 5.152, 95% CI 1.936–13.711, *P* = 0.001), involvement in a cluster (HR 0.365, 95% CI 0.180–0.743, *P* = 0.005), any symptom during the acute phase of Covid-19 (HR 0.368, 95% CI 0.161–0.842, *P* = 0.018), and pneumonia (HR 0.477, 95% CI 0.261–0.873, *P* = 0.016) were significantly associated with IgM seroconversion time. In contrast, age ≥ 40 years (HR 0.074, 95% CI 0.012–0.442, *P* = 0.004) was significantly associated with conversion from antiviral IgM positive to negative. No factor, however, was significantly associated with IgG duration in univariate proportional hazard model analyses (Table 3).

**Table 3:** Association between seroconversion and duration of IgG and IgM antibodies against SARS-CoV-2

	Generalized log-rank test		Proportional hazards models^¶^	
	Time interval^#^, Median (IQR)	*P* value	Hazard ratio (95%CI)	*P* value
IgG seroconversion				
Abroad imported case: yes	27 (19,35)	0.016*	6.331 (1.56–26.63)	0.010*
No	61 (58,82.5)			
Fever: yes	60 (53,77)	0.075	0.450 (0.204–0.993)	0.048^*^
No	27.5 (20.5,54.5)			
IgM seroconversion				
Abroad imported case: yes	26.5 (21.5,33)	0.001**	5.152 (1.936–13.711)	0.001**
No	61 (58,90)			
Involved in a cluster: yes	61 (59,150)	0.008**	0.365 (0.180–0.743)	0.005**
No	42 (26,62)			
Any symptom: yes	59.5 (32,89)	0.039*	0.368 (0.161–0.842)	0.018^*^
No	27.5 (22,53)			
Pneumonia: yes	60 (30,90)	0.015*	0.477 (0.261–0.873)	0.016*
No	53 (26,62)			
IgM duration^#^				
Age: ≥ 40 years	89 (30,122)	0.004**	0.074 (0.012–0.442)	0.004**
< 40 years	6 (5,6.5)			

Each serological result of patients was regarded as interval censored

*P < 0.05; **P < 0.01

^¶^Only statistically significant results are shown in the table

^#^Time interval for seroconversion means the interval from illness onset to first positive test for antibody. Time interval for duration means the interval from first to last positive antibody test

### Dynamics of peripheral lymphocytes

GEE model analyses suggested that T cell counts (β = − 0.8117, *P* = 0.0191), CD8^+^ T cell counts (β = − 0.414, *P* < 0.001) and CD4^+^/CD8^+^ ratio (β = − 0.0006, *P* = 0.002) decreased significantly over time, whereas CD4^+^ T cell and B cell counts did not. Considering the temporal change of peripheral lymphocytes in GEE models, antiviral IgM was significantly associated with T cell (β = − 147.173, *P* = 0.011), CD4^+^ T cell (β = − 83.74, *P* = 0.009), CD8^+^ T cell (β = − 51.32, *P* = 0.033), and B cell (β = − 35.50, *P* = 0.017) counts, whereas antiviral IgG was not significantly associated with any of these subsets of peripheral lymphocytes. Cigarette smoking was significantly associated with T cell (β = − 280.368, *P* = 0.0143) and CD4+ T cell (β = − 162.46, *P* = 0.0195) counts. Age ≥ 40 years was significantly associated with CD8+ T cell (β = − 129.687, *P* = 0.0016) and B cell (β = − 57.9, *P* = 0.0244) counts and with CD4+/CD8+ T cell ratios (β = 0.2686, *P* = 0.0215). Time from onset to admission (days) was significantly associated with CD8+ T cell counts (β = − 5.7616, *P* = 0.0191), and CD4+/CD8+ T cell ratio was significantly associated with male sex (β = − 0.2881, *P* = 0.0121).

### Self-reported symptoms during follow-up

A total of 1420 questionnaires were collected from the 168 face-to-face and 259 telephone participants over the 9 month follow-up period. Among these 1420 questionnaire interviews, 20 (1.4%), 18 (1.3%), 15 (1.1%), 11 (0.8%), 9 (0.6%), 9 (0.6%), 8 (0.6%), 6 (0.4%), 6 (0.4%), 5 (0.4%), 5 (0.4%), 4 (0.3%) and 71 (5%) reported fatigue, cough, shortness of breath, chest pain, fever, sputum production, joint pain, nasal congestion, runny nose, headache, muscle soreness, throat soreness, and at least one of the above symptoms, respectively. Self-reported shortness of breath was associated with clinical intensity, with all 15 patients who reported shortness of breath having normal clinical intensity (*P* = 0.037).

## Discussion

This 9-month prospective cohort study of patients in Shanghai with confirmed Covid-19 assessed long-term temporal changes in IgG and IgM antibodies against SARS-CoV-2 and identified factors associated with these changes. All 157 patients in the study cohort had mild or normal clinical intensity, the most common state in patients with Covid-19. To our knowledge, this is one of the limited long-term follow-up studies assessing the immune response against SARS-CoV-2 after discharge from the hospital of patients confirmed as having Covid-19.

The immune response patterns against SARS-CoV-2 observed in this study were similar to those against other coronaviruses, with strong immunity during the first 9 months after onset. IgG was detected 5 months [20] and 1 year [21] after infection with MERS-CoV, and the positivity rate remained stable, at 100%, during the first 16 months after SARS-CoV infection [22]. Antibodies against SARS-CoV provided patients with up to 2–3 years protection against re-infection [22, 23].

The present study observed that the antiviral IgG seropositive rate remained high (above 85%) for 2–9 months after disease onset and was 90.9% after 9 months. In addition, these IgG antibodies were present in 90.1% and 75.1% of participants 220 and 254 days, respectively, after seroconversion. These results showed that humoral immunity against SARS-CoV-2 was long-lived, with no decrease in IgG against SARS-CoV-2 for at least for 9 months after illness onset, in contrast to studies reporting a rapid reduction in antiviral antibodies immunity [9, 10]. Other studies have found that antibodies against SARS-CoV-2 remained stable for 4 months after diagnosis [24]; that more than 90% of seroconverters make detectable neutralizing antibodies and remained relatively stable for at least 5 months [25]; and that antibody titers declined but did not disappear for several months [26]. IgG can neutralize virus and plays an important role in long-term antiviral immunity following infection or vaccination [14, 24, 27]. Previous infection generating antibodies to SARS-CoV-2 was associated with protection from reinfection in most people for at least 6 months [13], with about 95% of subjects retaining immune memory for at least 6 months after infection [28]. The results of the present study indicated that the duration of vaccine-mediated immunity may be longer than previously reported. A longer duration of natural immunity, accompanied by reduced susceptibility [8], would therefore mitigate the pandemic.

SARS-CoV-2 has been shown to induce a classic immune response pattern to viral infection [14], with antiviral IgM increasing rapidly soon after onset and falling rapidly thereafter [12, 29] and IgG remaining detectable for several months [30]. The highest IgM seropositivity rate was observed within 1 month after onset, consistent with previous studies [31, 32]. The IgG seropositivity rate peaked 2 months after onset, later than previously reported [14, 31, 33]. Using survival analysis that treats serological observation as interval censored, both our study and a previous study [18] estimated an earlier and higher cumulative seroprevalence of IgG than IgM 1 month after onset, findings that differed from those of other studies [31, 32]. Because the time and sequence of seroconversion of antibody classes are important for prompt diagnosis of Covid-19, additional studies are needed to clarify these discrepancies.

The present study found that the seropositivity rate was higher in patients aged ≥ 40 than < 40 years, confirming that anti-SARS-CoV-2 antibody levels are higher in older people [29, 34–36]. However the mechanism is still unclear. We speculate that this may be due to the delayed immune response to SARS-CoV-2 in older people, slow in both production and disappear of antibody, which can explain more severe patients and higher seropositivity rate in older people. And age-related decline and dysregulation of immune function in older adults was hypothesized in literature [37]. Smokers tend to have lower IgG seropositivity rates than non-smokers, likely because smoking increases the expression of cellular receptors for entry of SARS-CoV-2 [38]. However, the association between smoking and immune response has been inconclusive [39, 40], which need more in-depth researches. Seropositivity was not significantly associated with gender, BMI, or clinical intensity.

The dynamics of peripheral lymphocytes have been described in patients recovering from Covid-19. T cell and CD8^+^ T cell counts decreased 9 months after disease onset, whereas CD4^+^ T cell and B cell counts did not. The observed trend of peripheral lymphocytes was consistent with that of SARS-CoV specific lymphocytes at 6–8 months, with these lymphocytes, along with antibodies, being responsible for immunological memory [28]. Our findings confirm that immunological memory responsible for functional antiviral immunity last more than 6 months after SARS-CoV infection [28, 41, 42].

Of the patients interviewed 9 months after infection, only 5% reported symptoms. In contrast, 76% of patients reported at least one symptom 6 months after disease onset [12]. This discrepancy may be due in part to differences in clinical intensity in the patient cohorts. All participants in the present study had mild or normal disease intensity, compared with only 25% in the previous study.

Colloidal gold immunoassays were used in the present study to assay for IgM and IgG antibodies against SARS-CoV-2. Although this is a point-of-care test applying lateral flow immunochromatographic methods, it has been recommended in research settings by the WHO [17]. Additionally, to improve the stability of results, all blood samples were tested in one laboratory by the same trained laboratory staff members using assay kits from a single manufacturer. Assay types have shown slight differences in sensitivity [16], with colloidal gold immunoassays showing good performance when compared with chemiluminescent microparticle immunoassays [43]. The finding of this study was similar to some studies using conventional immunological test [44, 45]. This indicates the rapid serological diagnostic test is a potentially important method in both surveillance and diagnosis to mitigate emerging and re-emerging pandemics of Covid-19 [46, 47]. And the clinical and scientific use of anti-IgM and anti-IgG antibodies in COVID-19 diagnosis should be highlighted.

This study had several limitations. First, the cohort population consisted only of participants with mild or normal clinical intensity of Covid-19, as few patients in Shanghai had severe or critical clinical intensity. Second, patients discharged within 7 days were excluded in this study, as the first follow-up visit began 7 days after the patient was discharge. This may bias our results when the assessment of short-term immune responses. And this study was more concerned with the long-term immune response. Third, antibodies against SARS-CoV-2 were evaluated qualitatively, not by assessing quantitative antibody titers. This may reduce the accuracy of temporal trends in antibody responses. Fourth, the number of participants was limited and the follow-up compliance was moderate, with 44–46 samples collected at each follow-up time from 157 participants.

## Conclusions

In conclusion, IgG antibody against SARS-CoV-2 in patients with a history of natural infection was long-lived, being detectable for at least 9 months after illness onset. In contrast, antiviral IgM antibody gradually declined after 1 month. Immune responses tended to be weaker in younger than in older patients and in smokers than in non-smokers. The long duration of natural immunity may have a positive impact on mitigation and elimination of the ongoing pandemic.

## Data Availability

The datasets supporting the conclusions of this article are available from the corresponding author on reasonable request.

## Appendix

See Table 4.

**Table 4:** Demographic, epidemiological and clinical characteristics of confirmed Covid-19 patients

Characteristics	Total confirmed patients (n = 1390)	Face-to-face follow-up patients (n = 157)	Telephone follow-up patients (n = 1233)	*P* value
Age (years)				0.917
0–39	818 (58.9)	93 (59.2)	725(58.8)	
40–89	572 (41.2)	64 (40.8)	508 (41.2)	
Gender				0.352
Male	853 (61.4)	91 (58.0)	762 (61.8)	
Occupation				< 0.001**
Students or children	277 (19.9)	30 (19.1)	247 (20.0)	
Retirees	179 (12.9)	28 (17.8)	151 (12.3)	
Civilian staff	259 (18.6)	53 (33.8)	206 (16.7)	
Other occupations	675 (48.6)	46 (29.3)	629 (51.0)	
Healthcare workers	8 (0.6)	3 (1.9)	5 (0.4)	0.019*
Pregnant women	7(1.3)	2(3.0)	5(1.1)	0.187
BMI (kg/m^2^)				0.048*
< 24.0	499 (59.1)	80 (52.0)	419 (60.6)	
≥ 24.0	346 (41.0)	74 (48.1)	272 (39.4)	
Residency				< 0.001**
Within Shanghai	232 (16.7)	65 (41.4)	167 (13.5)	
Outside Shanghai	947 (68.1)	85 (54.1)	862 (69.9)	
Foreign born	211 (15.2)	7 (4.5)	204 (16.5)	
Cigarette smoking	112 (13.0)	18 (12.2)	94 (13.1)	0.750
Alcohol drinking	197 (23.0)	45 (30.4)	152 (21.4)	0.018^*^
Comorbidities				
Diabetes	44 (5.0)	7 (4.6)	37 (5.0)	0.815
High blood pressure	86 (10.6)	17 (13.5)	69 (10.0)	0.243
Heart disease	27 (3.1)	3 (2.0)	24 (3.3)	0.392
Abroad imported case	1041 (74.9)	61 (38.9)	980 (79.5)	< 0.001**
Involved in a cluster	381 (27.4)	48 (30.6)	333 (27.0)	0.3456
Clinical intensity				< 0.001**
Mild	657 (47.3)	49 (31.2)	608 (49.3)	
Normal	725 (52.2)	108 (68.8)	617 (50.0)	
Severe or critical	8 (0.6)	0 (0.0)	8 (0.7)	
Duration from onset to admission, days	2.0 [1.0, 6.0]	4.0 [2.0, 7.0]	2.0 [1.0, 6.0]	0.002**
Duration from admission to discharge, days	13.0 [10.0, 19.0]	14.0 [10.0, 21.0]	13.0 [10.0, 18.0]	0.031*

Continuous variables are reported as median [IQR] and categorical variables as number (percentage)

**P* < 0.05; ***P* < 0.01

## Abbreviations

Covid-19: Coronavirus disease 2019
WHO: World Health Organization
IgG: Immunoglobulin G
IgM: Immunoglobulin M
SARS-CoV-2: Severe acute respiratory syndrome coronavirus 2
PHEIC: Public Health Emergency of International Concern
rRT-PCR: Real-time reverse-transcriptase polymerase chain-reaction
IQRs: Interquartile ranges
GEE: Generalized estimating equations
aOR: Adjusted odds ratio
HR: Hazard ratio
